# Bis(tetraphenylphosphonium) tetra­cyanido­nitridochromate(V) dihydrate

**DOI:** 10.1107/S1600536811002108

**Published:** 2011-01-22

**Authors:** Magnus Schau-Magnussen, Jesper Bendix

**Affiliations:** a Department of Chemistry, University of Copenhagen, Universitetsparken 5, DK-2100 Copenhagen, Denmark

## Abstract

In the title compound, (C_24_H_20_P)_2_[Cr(CN)_4_(N)]·2H_2_O, the complex anion exhibits a square-based pyramidal geometry around the central Cr^V^ atom, which is coordinated by a nitride ligand in the apical position and by four cyanide ligands in the equatorial plane. The chromium atom is located 0.4493 (13) Å out of the plane formed by the ligating C atoms of the cyanide ligands. The water mol­ecules of crystallization form inter­molecular O—H⋯N hydrogen bonds to the N atoms of two cyanide ligands of neighbouring complex anions, forming an infinite hydrogen-bonded chain parallel to [011] of water mol­ecules and [Cr(N)(CN)_4_]^2−^ anions. The terminal nitride ligands are not engaged in inter­molecular inter­actions.

## Related literature

For related structures of nitridocyanidometalates, see: Baldas *et al.* (1990 ▶); Bendix *et al.* (1998 ▶, 2000 ▶); Britten *et al.* (1993 ▶); Che *et al.* (1989 ▶); Purcell *et al.* (1991 ▶); van der Westhuizen *et al.* (1994 ▶). For general background to Cr^V^ nitrido complexes, see: Birk & Bendix (2003 ▶).
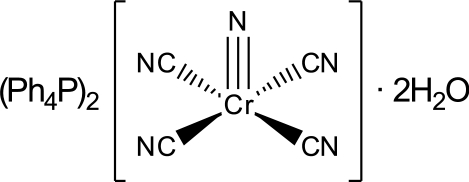


## Experimental

### 

#### Crystal data

(C_24_H_20_P)_2_[Cr(CN)_4_(N)]·2H_2_O
*M* *_r_* = 884.86
Triclinic, 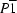

*a* = 11.996 (5) Å
*b* = 12.387 (5) Å
*c* = 16.721 (4) Å
α = 98.34 (3)°
β = 110.01 (2)°
γ = 90.52 (4)°
*V* = 2305.3 (15) Å^3^
*Z* = 2
Mo *K*α radiation
μ = 0.36 mm^−1^
*T* = 122 K
0.12 × 0.09 × 0.06 mm

#### Data collection

Nonius KappaCCD area-detector diffractometer
Absorption correction: integration (Gaussian; Coppens, 1970 ▶) *T*_min_ = 0.952, *T*_max_ = 0.980
72047 measured reflections
8118 independent reflections
6480 reflections with *I* > 2σ(*I*)
*R*_int_ = 0.061

#### Refinement

*R*[*F* ^2^ > 2σ(*F* ^2^)] = 0.039
*wR*(*F* ^2^) = 0.092
*S* = 1.10
8118 reflections
571 parameters
4 restraints
H atoms treated by a mixture of independent and constrained refinement
Δρ_max_ = 0.41 e Å^−3^
Δρ_min_ = −0.44 e Å^−3^

### 

Data collection: *COLLECT* (Nonius, 1999 ▶); cell refinement: *COLLECT*; data reduction: *EVALCCD* (Duisenberg *et al.*, 2003 ▶); program(s) used to solve structure: *SHELXS97* (Sheldrick, 2008 ▶); program(s) used to refine structure: *SHELXL97* (Sheldrick, 2008 ▶); molecular graphics: *ORTEP-3* (Farrugia, 1997 ▶); software used to prepare material for publication: *SHELXL97*.

## Supplementary Material

Crystal structure: contains datablocks global, I. DOI: 10.1107/S1600536811002108/fj2383sup1.cif

Structure factors: contains datablocks I. DOI: 10.1107/S1600536811002108/fj2383Isup2.hkl

Additional supplementary materials:  crystallographic information; 3D view; checkCIF report

## Figures and Tables

**Table:** 

Cr—N1	1.538 (2)
Cr—C2	2.066 (3)
Cr—C3	2.040 (3)
Cr—C4	2.068 (3)
Cr—C5	2.049 (3)

**Table:** 

N1—Cr—C2	99.23 (11)
N1—Cr—C3	105.79 (11)
N1—Cr—C4	99.95 (11)
N1—Cr—C5	105.70 (11)

**Table 2:** Hydrogen-bond geometry (Å, °)

*D*—H⋯*A*	*D*—H	H⋯*A*	*D*⋯*A*	*D*—H⋯*A*
O1—H1*A*⋯N2	0.86 (2)	2.23 (2)	3.065 (3)	167 (4)
O1—H1*B*⋯N2^i^	0.85 (2)	2.18 (2)	3.035 (3)	177 (4)
O2—H2*A*⋯N4	0.81 (2)	2.16 (2)	2.973 (3)	178 (3)
O2—H2*B*⋯N4^ii^	0.82 (2)	2.22 (2)	3.039 (3)	173 (3)

Symmetry codes: (i) 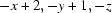; (ii) 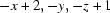.

**Table 3:** Comparative geometric parameters (Å) of cyanidonitridometalates of chromate(V) and manganate(V)

	[Cr(CN)_4_(N)]^2−*a*^	[Mn(CN)_4_(N)]^2−*b*^	[Cr(CN)_5_(N)]^3−*c*^	[Mn(CN)_5_(N)]^3−*c*^
*M* N	1.538 (2)	1.507 (2)	1.594 (9)	1.499 (8)
*M*—C*_*cis*_*	2.040 (3)–2.068 (3)	1.974 (2)–1.995 (2)	2.039 (7)–2.08 (2)	1.985 (6)–2.001 (7)
*M*—C*_*trans*_*			2.299 (12)	2.243 (7)
*M*—oop*^*d*^*	0.449	0.436	0.255	0.222

Notes: (*a*) this work; (*b*) Bendix *et al.* (1998 ▶); (*c*) Bendix *et al.* (2000 ▶); (*d*) oop = out-of-plane.

**Fig. 1.**
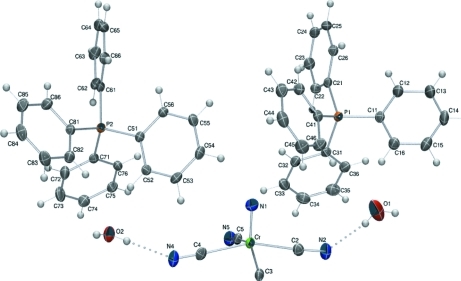
A view of the molecular structure of (1) with the atom-labelling scheme. Displacement ellipsoids are drawn at the 50% probability level.

**Fig. 2.**
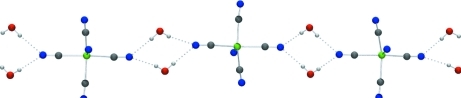
A ball-and-stick representation of the infinite chain of hydrogen bonded water molecules and [Cr(N)(CN)4]2- anions.

## supplementary crystallographic information

### Comment

Crystal structures of mononuclear nitridocyanidometalates have previously been reported for technetium (Baldas *et al.*, 1990), rhenium (Britten *et al.*, 1993; Purcell *et al.*, 1991), osmium (Che *et al.*, 1989; van der Westhuizen *et al.*, 1994). We have previously reported the syntheses and crystal stuctures of six coordinated pentacyanidonitridometalates of chromate(V) and manganate(V) (Bendix *et al.*, 2000) as well as the synthesis of the title compound and crystal structure of the isostructural manganese compound (Bendix *et al.*, 1998). In all the cases the nitride ligand imposes a strong *trans*-influence on the auxiliary ligands evidenced by a significant elongation of the M—*X_trans_* bond length and the displacement of the metal out of the plane spanned by cyanido-carbon atoms.

The molecular structure of the title compound is shown in Fig. 1. The Cr^V^ has a square based pyramidal coordination geometry frequently seen for Cr^V^ nitrido complexes (Birk & Bendix, 2003). The nitride occupy the apical position and the four cyanide ligands span the equatorial plane. The Cr atom is displaced 0.4493 (13) Å out of the plane spanned by the cyanido-carbon atoms. Selected geometric parameters are listed in Table 1. The water of crystallization form weak intermolecular hydrogen bonds (Table 2) to two nitrogen atoms of two cyanide ligands as depicted in Fig. 2. The complex anions and water molecules form an infinite chain. For comparison, Table 3 lists selected geometric parameters of the isostructural manganese compound and the six coordinated pentacyanidonitridometalates of chromate(V) and manganate(V).

### Experimental

The title compound was prepared as previously reported (Bendix *et al.*, 1998). A solution of [Cr(N)(CN)_4_]^2-^ (0.10 g; 0.21 mmol) in water (6.5 ml) was allowed to slowly diffuse into a solution of [PPh_4_]Cl (0.20 g; 0.53 mmol) in water (15 ml). The pale yellow crystals that precipitated were collected by filtration, washed with water, and air-dried. Yield: 0.16 g (86%).

### Refinement

H atoms on the phenyl groups were found in a difference Fourier map and were included in the refinement as constrained idealized protons riding the parent atom, with C—H = 0.95 Å. Ihe H atoms of the crystal waters were found in a difference Fourier map and were refined semi-free with a distance restraint and the U_iso_ equal to 1.5 times the U_eq_ of the parent oxygen.

### Crystal data

**Table:**

(C_24_H_20_P)_2_[Cr(CN)_4_(N)]·2H_2_O	*Z* = 2
*M**_r_* = 884.86	*F*(000) = 922
Triclinic, *P*1	*D*_x_ = 1.275 Mg m^−^^3^
Hall symbol: -P 1	Mo *K*α radiation, λ = 0.71073 Å
*a* = 11.996 (5) Å	Cell parameters from 83863 reflections
*b* = 12.387 (5) Å	θ = 1.3–27.6°
*c* = 16.721 (4) Å	µ = 0.36 mm^−^^1^
α = 98.34 (3)°	*T* = 122 K
β = 110.01 (2)°	Prism, yellow
γ = 90.52 (4)°	0.12 × 0.09 × 0.06 mm
*V* = 2305.3 (15) Å^3^	

### Data collection

**Table:**

Nonius KappaCCD area-detector diffractometer	8118 independent reflections
Radiation source: fine-focus sealed tube	6480 reflections with *I* > 2σ(*I*)
graphite	*R*_int_ = 0.061
ω and φ scans	θ_max_ = 25.0°, θ_min_ = 1.3°
Absorption correction: integration (Gaussian; Coppens, 1970)	*h* = −14→14
*T*_min_ = 0.952, *T*_max_ = 0.980	*k* = −14→14
72047 measured reflections	*l* = −19→19

### Refinement

**Table:**

Refinement on *F*^2^	Primary atom site location: structure-invariant direct methods
Least-squares matrix: full	Secondary atom site location: difference Fourier map
*R*[*F*^2^ > 2σ(*F*^2^)] = 0.039	Hydrogen site location: inferred from neighbouring sites
*wR*(*F*^2^) = 0.092	H atoms treated by a mixture of independent and constrained refinement
*S* = 1.10	*w* = 1/[σ^2^(*F*_o_^2^) + (0.*P*)^2^ + 2.4326*P*] where *P* = (*F*_o_^2^ + 2*F*_c_^2^)/3
8118 reflections	(Δ/σ)_max_ = 0.001
571 parameters	Δρ_max_ = 0.41 e Å^−^^3^
4 restraints	Δρ_min_ = −0.44 e Å^−^^3^

### Special details

**Table:**

Geometry. All e.s.d.'s (except the e.s.d. in the dihedral angle between two l.s. planes) are estimated using the full covariance matrix. The cell e.s.d.'s are taken into account individually in the estimation of e.s.d.'s in distances, angles and torsion angles; correlations between e.s.d.'s in cell parameters are only used when they are defined by crystal symmetry. An approximate (isotropic) treatment of cell e.s.d.'s is used for estimating e.s.d.'s involving l.s. planes.Least-squares planes (*x*,*y*,*z* in crystal coordinates) and deviations from them (* indicates atom used to define plane)2.5074 (0.0102) *x* + 8.4452 (0.0095) *y* + 7.9575 (0.0134) *z* = 6.0445 (0.0089)* 0.1022 (0.0012) C2 * -0.1077 (0.0012) C3 * 0.1077 (0.0012) C4 * -0.1021 (0.0012) C5 0.4493 (0.0013) CrRms deviation of fitted atoms = 0.1050
Refinement. Refinement of *F*^2^ against ALL reflections. The weighted *R*-factor *wR* and goodness of fit *S* are based on *F*^2^, conventional *R*-factors *R* are based on *F*, with *F* set to zero for negative *F*^2^. The threshold expression of *F*^2^ > σ(*F*^2^) is used only for calculating *R*-factors(gt) *etc*. and is not relevant to the choice of reflections for refinement. *R*-factors based on *F*^2^ are statistically about twice as large as those based on *F*, and *R*- factors based on ALL data will be even larger.

### Fractional atomic coordinates and isotropic or equivalent isotropic displacement parameters (Å^2^)

**Table:**

	*x*	*y*	*z*	*U*_iso_*/*U*_eq_	
Cr	0.97125 (3)	0.24895 (3)	0.24580 (2)	0.01723 (10)	
N1	1.03216 (19)	0.34819 (17)	0.31458 (13)	0.0287 (5)	
C2	0.9557 (2)	0.3156 (2)	0.13640 (16)	0.0229 (5)	
N2	0.9541 (2)	0.35860 (19)	0.07957 (15)	0.0356 (6)	
C3	1.1055 (2)	0.16264 (19)	0.22511 (14)	0.0194 (5)	
N3	1.17809 (19)	0.10990 (18)	0.21318 (14)	0.0287 (5)	
C4	0.9671 (2)	0.1357 (2)	0.32438 (15)	0.0213 (5)	
N4	0.96893 (19)	0.07573 (18)	0.37107 (14)	0.0302 (5)	
C5	0.7926 (2)	0.25946 (19)	0.22163 (15)	0.0202 (5)	
N5	0.6916 (2)	0.25833 (18)	0.20697 (14)	0.0308 (5)	
P1	0.66573 (5)	0.81476 (5)	0.15855 (4)	0.01645 (14)	
C11	0.7218 (2)	0.88033 (19)	0.08928 (14)	0.0197 (5)	
C12	0.7143 (3)	0.9925 (2)	0.08778 (17)	0.0309 (6)	
H12	0.6772	1.0353	0.1223	0.037*	
C13	0.7613 (3)	1.0411 (2)	0.03558 (18)	0.0405 (7)	
H13	0.7575	1.1176	0.0351	0.049*	
C14	0.8135 (3)	0.9788 (2)	−0.01559 (17)	0.0344 (7)	
H14	0.8452	1.0125	−0.0514	0.041*	
C15	0.8200 (2)	0.8679 (2)	−0.01495 (16)	0.0292 (6)	
H15	0.8559	0.8254	−0.0505	0.035*	
C16	0.7746 (2)	0.8180 (2)	0.03735 (16)	0.0257 (6)	
H16	0.7794	0.7415	0.0378	0.031*	
C21	0.5901 (2)	0.91094 (18)	0.20943 (14)	0.0172 (5)	
C22	0.4676 (2)	0.89460 (19)	0.18939 (15)	0.0209 (5)	
H22	0.4259	0.8310	0.1516	0.025*	
C23	0.4068 (2)	0.9710 (2)	0.22452 (16)	0.0244 (6)	
H23	0.3235	0.9597	0.2109	0.029*	
C24	0.4674 (2)	1.0636 (2)	0.27944 (15)	0.0246 (6)	
H24	0.4255	1.1160	0.3035	0.030*	
C25	0.5885 (2)	1.08050 (19)	0.29953 (15)	0.0242 (6)	
H25	0.6293	1.1445	0.3372	0.029*	
C26	0.6512 (2)	1.00486 (19)	0.26518 (14)	0.0201 (5)	
H26	0.7346	1.0166	0.2793	0.024*	
C31	0.5587 (2)	0.70555 (18)	0.09688 (14)	0.0183 (5)	
C32	0.5339 (2)	0.62444 (19)	0.13962 (15)	0.0225 (5)	
H32	0.5833	0.6205	0.1972	0.027*	
C33	0.4381 (2)	0.5503 (2)	0.09839 (17)	0.0271 (6)	
H33	0.4211	0.4956	0.1276	0.032*	
C34	0.3670 (2)	0.5554 (2)	0.01483 (17)	0.0305 (6)	
H34	0.3006	0.5044	−0.0132	0.037*	
C35	0.3914 (2)	0.6341 (2)	−0.02869 (17)	0.0316 (6)	
H35	0.3425	0.6363	−0.0866	0.038*	
C36	0.4872 (2)	0.7097 (2)	0.01209 (15)	0.0243 (6)	
H36	0.5040	0.7640	−0.0176	0.029*	
C41	0.7823 (2)	0.75651 (19)	0.23712 (15)	0.0193 (5)	
C42	0.8155 (2)	0.7932 (2)	0.32521 (15)	0.0237 (5)	
H42	0.7795	0.8538	0.3458	0.028*	
C43	0.9016 (2)	0.7406 (2)	0.38291 (16)	0.0290 (6)	
H43	0.9252	0.7662	0.4430	0.035*	
C44	0.9530 (2)	0.6517 (2)	0.35352 (17)	0.0284 (6)	
H44	1.0107	0.6155	0.3934	0.034*	
C45	0.9207 (2)	0.6150 (2)	0.26603 (17)	0.0297 (6)	
H45	0.9566	0.5538	0.2460	0.036*	
C46	0.8365 (2)	0.6671 (2)	0.20761 (16)	0.0276 (6)	
H46	0.8152	0.6423	0.1475	0.033*	
P2	0.65911 (5)	0.34699 (5)	0.62208 (4)	0.01508 (13)	
C51	0.72980 (19)	0.38164 (18)	0.54899 (14)	0.0158 (5)	
C52	0.7530 (2)	0.29675 (19)	0.49283 (14)	0.0183 (5)	
H52	0.7296	0.2232	0.4926	0.022*	
C53	0.8102 (2)	0.3205 (2)	0.43758 (14)	0.0217 (5)	
H53	0.8276	0.2629	0.4004	0.026*	
C54	0.8421 (2)	0.4279 (2)	0.43637 (15)	0.0224 (5)	
H54	0.8807	0.4439	0.3980	0.027*	
C55	0.8178 (2)	0.5120 (2)	0.49107 (15)	0.0231 (5)	
H55	0.8393	0.5856	0.4899	0.028*	
C56	0.7620 (2)	0.48910 (19)	0.54750 (14)	0.0182 (5)	
H56	0.7459	0.5470	0.5852	0.022*	
C61	0.6187 (2)	0.46906 (18)	0.67540 (14)	0.0179 (5)	
C62	0.4995 (2)	0.4868 (2)	0.66260 (15)	0.0220 (5)	
H62	0.4386	0.4351	0.6246	0.026*	
C63	0.4707 (2)	0.5803 (2)	0.70586 (16)	0.0287 (6)	
H63	0.3897	0.5925	0.6975	0.034*	
C64	0.5586 (3)	0.6560 (2)	0.76098 (16)	0.0303 (6)	
H64	0.5377	0.7200	0.7901	0.036*	
C65	0.6773 (2)	0.6389 (2)	0.77403 (16)	0.0281 (6)	
H65	0.7376	0.6912	0.8120	0.034*	
C66	0.7081 (2)	0.54541 (19)	0.73164 (15)	0.0238 (6)	
H66	0.7893	0.5333	0.7407	0.029*	
C71	0.5277 (2)	0.26263 (18)	0.55768 (14)	0.0169 (5)	
C72	0.4935 (2)	0.1720 (2)	0.58589 (16)	0.0252 (6)	
H72	0.5418	0.1514	0.6389	0.030*	
C73	0.3883 (2)	0.1116 (2)	0.53614 (17)	0.0314 (6)	
H73	0.3649	0.0494	0.5551	0.038*	
C74	0.3175 (2)	0.1417 (2)	0.45915 (16)	0.0264 (6)	
H74	0.2454	0.1002	0.4256	0.032*	
C75	0.3512 (2)	0.23212 (19)	0.43086 (15)	0.0221 (5)	
H75	0.3022	0.2528	0.3781	0.027*	
C76	0.4566 (2)	0.29250 (18)	0.47958 (14)	0.0189 (5)	
H76	0.4804	0.3540	0.4599	0.023*	
C81	0.75605 (19)	0.27572 (18)	0.70289 (14)	0.0164 (5)	
C82	0.8202 (2)	0.19116 (19)	0.67944 (16)	0.0232 (5)	
H82	0.8129	0.1710	0.6208	0.028*	
C83	0.8942 (2)	0.1368 (2)	0.74176 (17)	0.0276 (6)	
H83	0.9374	0.0789	0.7257	0.033*	
C84	0.9056 (2)	0.1663 (2)	0.82709 (17)	0.0283 (6)	
H84	0.9572	0.1289	0.8696	0.034*	
C85	0.8427 (2)	0.2498 (2)	0.85098 (16)	0.0302 (6)	
H85	0.8510	0.2699	0.9099	0.036*	
C86	0.7672 (2)	0.3046 (2)	0.78916 (15)	0.0247 (6)	
H86	0.7233	0.3617	0.8055	0.030*	
O1	0.8836 (2)	0.57434 (19)	0.01213 (19)	0.0611 (7)	
H1A	0.910 (4)	0.513 (2)	0.025 (3)	0.092*	
H1B	0.931 (3)	0.592 (3)	−0.013 (2)	0.092*	
O2	0.85439 (16)	0.04312 (15)	0.49856 (12)	0.0291 (4)	
H2A	0.884 (3)	0.052 (2)	0.4627 (16)	0.044*	
H2B	0.906 (2)	0.016 (2)	0.5356 (16)	0.044*	

### Atomic displacement parameters (Å^2^)

**Table:**

	*U*^11^	*U*^22^	*U*^33^	*U*^12^	*U*^13^	*U*^23^
Cr	0.0185 (2)	0.0193 (2)	0.0159 (2)	0.00301 (16)	0.00804 (16)	0.00416 (15)
N1	0.0316 (13)	0.0278 (12)	0.0253 (11)	−0.0007 (10)	0.0099 (10)	0.0000 (9)
C2	0.0230 (14)	0.0229 (13)	0.0254 (14)	0.0029 (11)	0.0108 (11)	0.0058 (11)
N2	0.0412 (14)	0.0395 (14)	0.0325 (13)	0.0045 (11)	0.0165 (11)	0.0162 (11)
C3	0.0204 (13)	0.0226 (13)	0.0177 (12)	−0.0011 (11)	0.0089 (10)	0.0053 (10)
N3	0.0271 (12)	0.0321 (12)	0.0328 (13)	0.0064 (10)	0.0162 (10)	0.0088 (10)
C4	0.0174 (13)	0.0286 (14)	0.0214 (13)	0.0058 (10)	0.0106 (11)	0.0049 (11)
N4	0.0309 (13)	0.0384 (13)	0.0302 (12)	0.0106 (10)	0.0168 (10)	0.0174 (11)
C5	0.0258 (15)	0.0203 (13)	0.0199 (12)	0.0081 (11)	0.0128 (11)	0.0073 (10)
N5	0.0293 (14)	0.0351 (13)	0.0353 (13)	0.0121 (10)	0.0174 (11)	0.0132 (10)
P1	0.0173 (3)	0.0175 (3)	0.0153 (3)	0.0030 (2)	0.0064 (3)	0.0029 (2)
C11	0.0192 (13)	0.0224 (13)	0.0177 (12)	0.0014 (10)	0.0057 (10)	0.0053 (10)
C12	0.0474 (18)	0.0236 (14)	0.0283 (14)	0.0048 (12)	0.0206 (13)	0.0061 (11)
C13	0.071 (2)	0.0251 (15)	0.0340 (16)	0.0009 (14)	0.0282 (16)	0.0087 (13)
C14	0.0440 (17)	0.0393 (17)	0.0251 (14)	−0.0057 (13)	0.0162 (13)	0.0110 (12)
C15	0.0295 (15)	0.0398 (16)	0.0215 (13)	0.0035 (12)	0.0128 (12)	0.0049 (12)
C16	0.0302 (15)	0.0236 (13)	0.0264 (14)	0.0061 (11)	0.0126 (12)	0.0066 (11)
C21	0.0202 (13)	0.0175 (12)	0.0158 (11)	0.0042 (10)	0.0078 (10)	0.0048 (9)
C22	0.0200 (13)	0.0202 (13)	0.0205 (12)	0.0010 (10)	0.0051 (10)	0.0020 (10)
C23	0.0196 (13)	0.0259 (14)	0.0308 (14)	0.0056 (11)	0.0121 (11)	0.0062 (11)
C24	0.0337 (15)	0.0202 (13)	0.0253 (13)	0.0082 (11)	0.0166 (12)	0.0047 (11)
C25	0.0343 (15)	0.0179 (13)	0.0199 (13)	−0.0009 (11)	0.0103 (11)	−0.0006 (10)
C26	0.0187 (13)	0.0215 (13)	0.0200 (12)	0.0003 (10)	0.0064 (10)	0.0040 (10)
C31	0.0186 (12)	0.0190 (12)	0.0171 (12)	0.0047 (10)	0.0066 (10)	0.0012 (10)
C32	0.0262 (14)	0.0235 (13)	0.0190 (12)	0.0038 (11)	0.0092 (11)	0.0039 (10)
C33	0.0290 (15)	0.0220 (13)	0.0331 (15)	−0.0011 (11)	0.0146 (12)	0.0042 (11)
C34	0.0225 (14)	0.0258 (14)	0.0373 (16)	−0.0020 (11)	0.0056 (12)	−0.0022 (12)
C35	0.0287 (15)	0.0303 (15)	0.0246 (14)	0.0033 (12)	−0.0034 (12)	0.0000 (12)
C36	0.0285 (14)	0.0226 (13)	0.0219 (13)	0.0047 (11)	0.0083 (11)	0.0042 (11)
C41	0.0163 (12)	0.0206 (12)	0.0207 (12)	0.0010 (10)	0.0048 (10)	0.0066 (10)
C42	0.0250 (14)	0.0232 (13)	0.0228 (13)	0.0014 (11)	0.0079 (11)	0.0040 (11)
C43	0.0285 (15)	0.0325 (15)	0.0220 (13)	−0.0030 (12)	0.0026 (12)	0.0071 (11)
C44	0.0166 (13)	0.0320 (15)	0.0354 (15)	−0.0001 (11)	0.0029 (12)	0.0163 (12)
C45	0.0246 (14)	0.0290 (15)	0.0360 (16)	0.0102 (12)	0.0096 (12)	0.0089 (12)
C46	0.0274 (14)	0.0309 (15)	0.0236 (13)	0.0084 (12)	0.0079 (12)	0.0034 (11)
P2	0.0148 (3)	0.0161 (3)	0.0153 (3)	0.0016 (2)	0.0065 (2)	0.0024 (2)
C51	0.0124 (11)	0.0202 (12)	0.0146 (11)	0.0021 (9)	0.0039 (9)	0.0040 (9)
C52	0.0186 (12)	0.0188 (12)	0.0168 (12)	0.0012 (10)	0.0051 (10)	0.0031 (10)
C53	0.0206 (13)	0.0290 (14)	0.0143 (12)	0.0045 (11)	0.0056 (10)	0.0009 (10)
C54	0.0188 (13)	0.0326 (14)	0.0178 (12)	0.0000 (11)	0.0082 (10)	0.0058 (11)
C55	0.0233 (13)	0.0227 (13)	0.0236 (13)	−0.0033 (11)	0.0075 (11)	0.0067 (11)
C56	0.0190 (12)	0.0193 (12)	0.0168 (12)	0.0016 (10)	0.0073 (10)	0.0018 (10)
C61	0.0233 (13)	0.0178 (12)	0.0165 (12)	0.0021 (10)	0.0115 (10)	0.0039 (10)
C62	0.0233 (13)	0.0266 (13)	0.0176 (12)	0.0068 (11)	0.0082 (11)	0.0054 (10)
C63	0.0305 (15)	0.0355 (15)	0.0245 (14)	0.0174 (12)	0.0128 (12)	0.0092 (12)
C64	0.0522 (19)	0.0199 (13)	0.0276 (14)	0.0136 (13)	0.0231 (14)	0.0083 (11)
C65	0.0439 (17)	0.0189 (13)	0.0265 (14)	−0.0050 (12)	0.0204 (13)	−0.0001 (11)
C66	0.0256 (14)	0.0240 (13)	0.0268 (14)	−0.0019 (11)	0.0159 (12)	0.0027 (11)
C71	0.0155 (12)	0.0185 (12)	0.0176 (12)	0.0016 (10)	0.0073 (10)	0.0018 (10)
C72	0.0256 (14)	0.0298 (14)	0.0209 (13)	−0.0021 (11)	0.0069 (11)	0.0094 (11)
C73	0.0296 (15)	0.0342 (15)	0.0309 (15)	−0.0099 (12)	0.0087 (12)	0.0120 (12)
C74	0.0217 (14)	0.0296 (14)	0.0256 (14)	−0.0056 (11)	0.0070 (11)	0.0009 (11)
C75	0.0212 (13)	0.0262 (13)	0.0170 (12)	0.0028 (11)	0.0044 (10)	0.0026 (10)
C76	0.0231 (13)	0.0156 (12)	0.0197 (12)	0.0019 (10)	0.0092 (11)	0.0039 (10)
C81	0.0139 (12)	0.0178 (12)	0.0171 (12)	−0.0013 (9)	0.0046 (10)	0.0037 (9)
C82	0.0266 (14)	0.0242 (13)	0.0213 (13)	0.0031 (11)	0.0106 (11)	0.0054 (10)
C83	0.0235 (14)	0.0271 (14)	0.0373 (15)	0.0080 (11)	0.0133 (12)	0.0141 (12)
C84	0.0190 (13)	0.0331 (15)	0.0306 (15)	−0.0014 (11)	0.0006 (11)	0.0178 (12)
C85	0.0351 (16)	0.0342 (15)	0.0156 (13)	−0.0035 (13)	0.0014 (12)	0.0046 (11)
C86	0.0286 (14)	0.0261 (14)	0.0186 (13)	0.0022 (11)	0.0078 (11)	0.0020 (11)
O1	0.0613 (16)	0.0420 (13)	0.112 (2)	0.0201 (12)	0.0621 (16)	0.0316 (14)
O2	0.0262 (10)	0.0365 (11)	0.0308 (11)	0.0065 (8)	0.0147 (9)	0.0133 (9)

### Geometric parameters (Å, °)

**Table:**

Cr—N1	1.538 (2)	C45—H45	0.9500
Cr—C2	2.066 (3)	C46—H46	0.9500
Cr—C3	2.040 (3)	P2—C71	1.794 (2)
Cr—C4	2.068 (3)	P2—C61	1.798 (2)
Cr—C5	2.049 (3)	P2—C81	1.802 (2)
C2—N2	1.150 (3)	P2—C51	1.803 (2)
C3—N3	1.147 (3)	C51—C56	1.389 (3)
C4—N4	1.148 (3)	C51—C52	1.401 (3)
C5—N5	1.150 (3)	C52—C53	1.385 (3)
P1—C31	1.789 (3)	C52—H52	0.9500
P1—C21	1.795 (2)	C53—C54	1.387 (3)
P1—C41	1.800 (2)	C53—H53	0.9500
P1—C11	1.803 (2)	C54—C55	1.385 (3)
C11—C16	1.394 (3)	C54—H54	0.9500
C11—C12	1.396 (3)	C55—C56	1.387 (3)
C12—C13	1.388 (4)	C55—H55	0.9500
C12—H12	0.9500	C56—H56	0.9500
C13—C14	1.380 (4)	C61—C62	1.396 (3)
C13—H13	0.9500	C61—C66	1.401 (3)
C14—C15	1.378 (4)	C62—C63	1.386 (3)
C14—H14	0.9500	C62—H62	0.9500
C15—C16	1.386 (3)	C63—C64	1.381 (4)
C15—H15	0.9500	C63—H63	0.9500
C16—H16	0.9500	C64—C65	1.389 (4)
C21—C22	1.396 (3)	C64—H64	0.9500
C21—C26	1.401 (3)	C65—C66	1.389 (3)
C22—C23	1.384 (3)	C65—H65	0.9500
C22—H22	0.9500	C66—H66	0.9500
C23—C24	1.382 (4)	C71—C72	1.389 (3)
C23—H23	0.9500	C71—C76	1.398 (3)
C24—C25	1.381 (4)	C72—C73	1.388 (4)
C24—H24	0.9500	C72—H72	0.9500
C25—C26	1.388 (3)	C73—C74	1.384 (4)
C25—H25	0.9500	C73—H73	0.9500
C26—H26	0.9500	C74—C75	1.385 (3)
C31—C36	1.392 (3)	C74—H74	0.9500
C31—C32	1.399 (3)	C75—C76	1.386 (3)
C32—C33	1.376 (4)	C75—H75	0.9500
C32—H32	0.9500	C76—H76	0.9500
C33—C34	1.376 (4)	C81—C86	1.394 (3)
C33—H33	0.9500	C81—C82	1.394 (3)
C34—C35	1.383 (4)	C82—C83	1.381 (3)
C34—H34	0.9500	C82—H82	0.9500
C35—C36	1.387 (4)	C83—C84	1.379 (4)
C35—H35	0.9500	C83—H83	0.9500
C36—H36	0.9500	C84—C85	1.379 (4)
C41—C42	1.391 (3)	C84—H84	0.9500
C41—C46	1.402 (3)	C85—C86	1.387 (4)
C42—C43	1.391 (4)	C85—H85	0.9500
C42—H42	0.9500	C86—H86	0.9500
C43—C44	1.377 (4)	O1—H1A	0.856 (19)
C43—H43	0.9500	O1—H1B	0.854 (19)
C44—C45	1.383 (4)	O2—H2A	0.814 (17)
C44—H44	0.9500	O2—H2B	0.824 (17)
C45—C46	1.383 (4)		
			
N1—Cr—C2	99.23 (11)	C46—C45—C44	120.2 (2)
N1—Cr—C3	105.79 (11)	C46—C45—H45	119.9
N1—Cr—C4	99.95 (11)	C44—C45—H45	119.9
N1—Cr—C5	105.70 (11)	C45—C46—C41	120.0 (2)
C3—Cr—C5	148.38 (10)	C45—C46—H46	120.0
C3—Cr—C2	87.56 (10)	C41—C46—H46	120.0
C5—Cr—C2	90.28 (10)	C71—P2—C61	109.65 (11)
C3—Cr—C4	84.71 (9)	C71—P2—C81	110.71 (11)
C5—Cr—C4	87.09 (10)	C61—P2—C81	108.47 (11)
C2—Cr—C4	160.64 (10)	C71—P2—C51	106.53 (11)
N2—C2—Cr	174.6 (2)	C61—P2—C51	110.02 (11)
N3—C3—Cr	177.0 (2)	C81—P2—C51	111.46 (11)
N4—C4—Cr	176.8 (2)	C56—C51—C52	119.7 (2)
N5—C5—Cr	175.7 (2)	C56—C51—P2	121.93 (17)
C31—P1—C21	106.70 (11)	C52—C51—P2	118.37 (17)
C31—P1—C41	107.08 (11)	C53—C52—C51	119.7 (2)
C21—P1—C41	111.23 (11)	C53—C52—H52	120.1
C31—P1—C11	110.46 (11)	C51—C52—H52	120.1
C21—P1—C11	109.57 (11)	C52—C53—C54	120.3 (2)
C41—P1—C11	111.66 (11)	C52—C53—H53	119.9
C16—C11—C12	119.8 (2)	C54—C53—H53	119.9
C16—C11—P1	119.24 (18)	C55—C54—C53	120.0 (2)
C12—C11—P1	120.99 (18)	C55—C54—H54	120.0
C13—C12—C11	119.6 (2)	C53—C54—H54	120.0
C13—C12—H12	120.2	C54—C55—C56	120.2 (2)
C11—C12—H12	120.2	C54—C55—H55	119.9
C14—C13—C12	120.3 (3)	C56—C55—H55	119.9
C14—C13—H13	119.9	C55—C56—C51	120.0 (2)
C12—C13—H13	119.9	C55—C56—H56	120.0
C15—C14—C13	120.3 (2)	C51—C56—H56	120.0
C15—C14—H14	119.8	C62—C61—C66	120.1 (2)
C13—C14—H14	119.8	C62—C61—P2	120.50 (18)
C14—C15—C16	120.3 (2)	C66—C61—P2	119.43 (18)
C14—C15—H15	119.9	C63—C62—C61	119.4 (2)
C16—C15—H15	119.9	C63—C62—H62	120.3
C15—C16—C11	119.8 (2)	C61—C62—H62	120.3
C15—C16—H16	120.1	C64—C63—C62	120.7 (2)
C11—C16—H16	120.1	C64—C63—H63	119.7
C22—C21—C26	119.8 (2)	C62—C63—H63	119.7
C22—C21—P1	119.34 (18)	C63—C64—C65	120.2 (2)
C26—C21—P1	120.78 (18)	C63—C64—H64	119.9
C23—C22—C21	120.1 (2)	C65—C64—H64	119.9
C23—C22—H22	120.0	C66—C65—C64	120.0 (2)
C21—C22—H22	120.0	C66—C65—H65	120.0
C24—C23—C22	120.0 (2)	C64—C65—H65	120.0
C24—C23—H23	120.0	C65—C66—C61	119.6 (2)
C22—C23—H23	120.0	C65—C66—H66	120.2
C25—C24—C23	120.3 (2)	C61—C66—H66	120.2
C25—C24—H24	119.9	C72—C71—C76	120.1 (2)
C23—C24—H24	119.9	C72—C71—P2	121.21 (18)
C24—C25—C26	120.7 (2)	C76—C71—P2	118.66 (17)
C24—C25—H25	119.7	C73—C72—C71	119.6 (2)
C26—C25—H25	119.7	C73—C72—H72	120.2
C25—C26—C21	119.2 (2)	C71—C72—H72	120.2
C25—C26—H26	120.4	C74—C73—C72	120.4 (2)
C21—C26—H26	120.4	C74—C73—H73	119.8
C36—C31—C32	119.7 (2)	C72—C73—H73	119.8
C36—C31—P1	121.13 (18)	C73—C74—C75	120.2 (2)
C32—C31—P1	118.33 (18)	C73—C74—H74	119.9
C33—C32—C31	120.0 (2)	C75—C74—H74	119.9
C33—C32—H32	120.0	C74—C75—C76	120.0 (2)
C31—C32—H32	120.0	C74—C75—H75	120.0
C32—C33—C34	120.1 (2)	C76—C75—H75	120.0
C32—C33—H33	120.0	C75—C76—C71	119.8 (2)
C34—C33—H33	120.0	C75—C76—H76	120.1
C33—C34—C35	120.5 (2)	C71—C76—H76	120.1
C33—C34—H34	119.7	C86—C81—C82	119.6 (2)
C35—C34—H34	119.7	C86—C81—P2	120.22 (18)
C34—C35—C36	120.1 (2)	C82—C81—P2	120.14 (17)
C34—C35—H35	120.0	C83—C82—C81	119.8 (2)
C36—C35—H35	120.0	C83—C82—H82	120.1
C35—C36—C31	119.5 (2)	C81—C82—H82	120.1
C35—C36—H36	120.2	C84—C83—C82	120.4 (2)
C31—C36—H36	120.2	C84—C83—H83	119.8
C42—C41—C46	119.5 (2)	C82—C83—H83	119.8
C42—C41—P1	122.46 (18)	C83—C84—C85	120.3 (2)
C46—C41—P1	117.92 (18)	C83—C84—H84	119.8
C43—C42—C41	119.6 (2)	C85—C84—H84	119.8
C43—C42—H42	120.2	C84—C85—C86	120.1 (2)
C41—C42—H42	120.2	C84—C85—H85	120.0
C44—C43—C42	120.5 (2)	C86—C85—H85	120.0
C44—C43—H43	119.8	C85—C86—C81	119.8 (2)
C42—C43—H43	119.8	C85—C86—H86	120.1
C43—C44—C45	120.2 (2)	C81—C86—H86	120.1
C43—C44—H44	119.9	H1A—O1—H1B	100 (4)
C45—C44—H44	119.9	H2A—O2—H2B	105 (3)

### Hydrogen-bond geometry (Å, °)

**Table:**

*D*—H···*A*	*D*—H	H···*A*	*D*···*A*	*D*—H···*A*
O1—H1A···N2	0.86 (2)	2.23 (2)	3.065 (3)	167 (4)
O1—H1B···N2^i^	0.85 (2)	2.18 (2)	3.035 (3)	177 (4)
O2—H2A···N4	0.81 (2)	2.16 (2)	2.973 (3)	178 (3)
O2—H2B···N4^ii^	0.82 (2)	2.22 (2)	3.039 (3)	173 (3)

Symmetry codes: (i) −*x*+2, −*y*+1, −*z*; (ii) −*x*+2, −*y*, −*z*+1.

### Table 3 Comparative geometric parameters (Å) of cyanidonitridometalates of chromate(V) and manganate(V).

**Table:**

	[Cr(CN)_4_(N)]^2-^*a*	[Mn(CN)_4_(N)]^2-^*^b^*	[Cr(CN)_5_(N)]^3-^*^c^*	[Mn(CN)_5_(N)]^3-^*^c^*
*M*≡N	1.538 (2)	1.507 (2)	1.594 (9)	1.499 (8)
*M*—C*_cis_*	2.040 (3)-2.068 (3)	1.974 (2)-1.995 (2)	2.039 (7)-2.08 (2)	1.985 (6)-2.001 (7)
*M*—C*_trans_*			2.299 (12)	2.243 (7)
*M*—oop*^d^*	0.449	0.436	0.255	0.222

Notes: (*a*) this work; (*b*) Bendix *et al.* (1998); (*c*) Bendix *et al.* (2000); (*d*) oop = out-of-plane.

## References

[bb1] Baldas, J., Boas, J. F., Colmanet, S. F. & Mackay, M. F. (1990). *Inorg. Chim. Acta*, **170**, 233–239.

[bb2] Bendix, J., Deeth, R. J., Weyhermüller, T., Bill, E. & Wieghardt, K. (2000). *Inorg. Chem.* **39**, 930–938. 10.1021/ic990971j 12526371

[bb3] Bendix, J., Meyer, K., Weyhermüller, T., Bill, E., Metzler-Nolte, N. & Wieghardt, K. (1998). *Inorg. Chem.* **37**, 1767–1775.

[bb4] Birk, T. & Bendix, J. (2003). *Inorg. Chem.* **42**, 7608–7615. 10.1021/ic034777f 14606858

[bb5] Britten, J. F., Lock, C. J. L. & Wei, Y. (1993). *Acta Cryst.* C**49**, 1277–1280.

[bb6] Che, C. M., Lam, H. W. & Mak, T. C. W. (1989). *J. Chem. Soc. Chem. Commun.* pp. 1529–1531.

[bb7] Coppens, P. (1970). *Crystallographic Computing*, edited by F. R. Ahmed, S. R. Hall & C. P. Huber, pp. 255–270. Copenhagen: Munksgaard.

[bb8] Duisenberg, A. J. M., Kroon-Batenburg, L. M. J. & Schreurs, A. M. M. (2003). *J. Appl. Cryst.* **36**, 220–229.

[bb9] Farrugia, L. J. (1997). *J. Appl. Cryst.* **30**, 565.

[bb10] Nonius (1999). *COLLECT* Nonius BV, Delft, The Netherlands.

[bb11] Purcell, W., Potgieter, I. Z., Damoense, L. J. & Leipolldt, J. S. (1991). *Transition Met. Chem.* **16**, 473–475.

[bb12] Sheldrick, G. M. (2008). *Acta Cryst.* A**64**, 112–122. 10.1107/S0108767307043930 18156677

[bb13] Westhuizen, H. J. van der, Basson, S. S., Leipoldt, J. G. & Purcell, W. (1994). *Transition Met. Chem.* **19**, 582–584.

